# The efficacy of tranexamic acid using oral administration in total knee arthroplasty: a systematic review and meta-analysis

**DOI:** 10.1186/s13018-017-0660-6

**Published:** 2017-10-27

**Authors:** Lu-kai Zhang, Jian-xiong Ma, Ming-jie Kuang, Jie Zhao, Bin Lu, Ying Wang, Xin-long Ma, Zheng-rui Fan

**Affiliations:** 1 Biomechanics Labs of Orthopaedics Institute, Tianjin Hospital, No. 155, Munan Road, Heping District, Tianjin, 300050 People’s Republic of China; 2 Graduate School of Tianjin University of Traditional Chinese Medicine, Tianjin, 300193 People’s Republic of China; 3 Department of Orthopedics, Tianjin Hospital, Tianjin, 300211 People’s Republic of China

**Keywords:** Tranexamic acid, Oral, Total knee arthroplasty, Blood loss, Meta-analysis

## Abstract

**Background:**

Total knee arthroplasty (TKA) is gradually regarded as an effective choice for end-stage osteoarthritis or rheumatic arthritis. In the past, the management of tranexamic acid (TXA) using intravenous injection or topical application has been extensively researched. However, several studies have reported that oral TXA has an effect on blood loss. Therefore, a meta-analysis should be performed to determine whether oral TXA helps to prevent blood loss.

**Methods:**

Randomized controlled trials or retrospective cohort studies about relevant studies were searched in PubMed (1996–April 2017), Embase (1980–April 2017), and the Cochrane Library (CENTRAL, April 2017). Six studies that compared oral TXA to non-TXA were included in our meta-analysis. Meta-analyses (PRISMA) guidelines, the Cochrane Handbook, and the Jadad scale were used to evaluate the included studies and the results to ensure that the meta-analysis was viable.

**Results:**

In accordance with inclusion and exclusion, six studies with 2553 patients (oral TXA = 1386, without TXA = 1167) were eligible and accepted into this meta-analysis. Pooled data indicated that the oral TXA group was effective compared to the without TXA group in terms of hemoglobin (Hb) drop (*P* < 0.05), blood loss at 24 h (*P* < 0.05), total blood loss (*P* < 0.05), and the transfusion rate (*P* < 0.05). No significant differences were found in the length of hospital stay (*P* = 0.96) and complications (*P* = 0.39).

**Conclusion:**

Compared to the non-TXA group, the oral TXA group showed effects of blood sparing. Considering the cost and effectiveness, oral TXA is useful for TKA.

## Background

Total knee arthroplasty (TKA) is recommended as an effective method for end-stage knee osteoarthritis or rheumatoid arthritis [1]. However, it may cause severe blood loss during the perioperative period. Tranexamic acid (TXA) is one of most important antifibrinolytic agents and is known to reduce blood loss, hemoglobin (Hb) drop, and blood transfusion [2–4]. While many meta-analyses and randomized controlled trials (RCTs) have focused on the efficacy of intravenous and topical TXA on TKA [5–7], few have demonstrated the safety and efficacy of oral administration [3, 8, 9]. While drug allergic reaction of anaphylactic shock have been reported in intravenous administration [10], topical administration has a risk of periprosthetic contamination due to infected needles that may cause septicopyemia [11]; in addition, the short duration of the topical form of the drug limits its application [11, 12]. Because oral TXA is administered gradually, a number of RCTs or retrospective controlled studies (RCSs) have been performed to investigate its efficacy and side effects [13–15]. Oral TXA application undergoes rapid and large absorption. Furthermore, it is easy to use without special equipment compared to intravenous or topical injection, is less costly, and offers a less burdensome workload for medical personnel [16]. No conclusive evidence has been found regarding the efficacy of using oral TXA during the perioperative period in TKA. The purpose of this meta-analysis was to determine the blood-sparing efficacy of oral TXA administration in TKA.

## Methods

### Search strategy

**Fig. 1:**
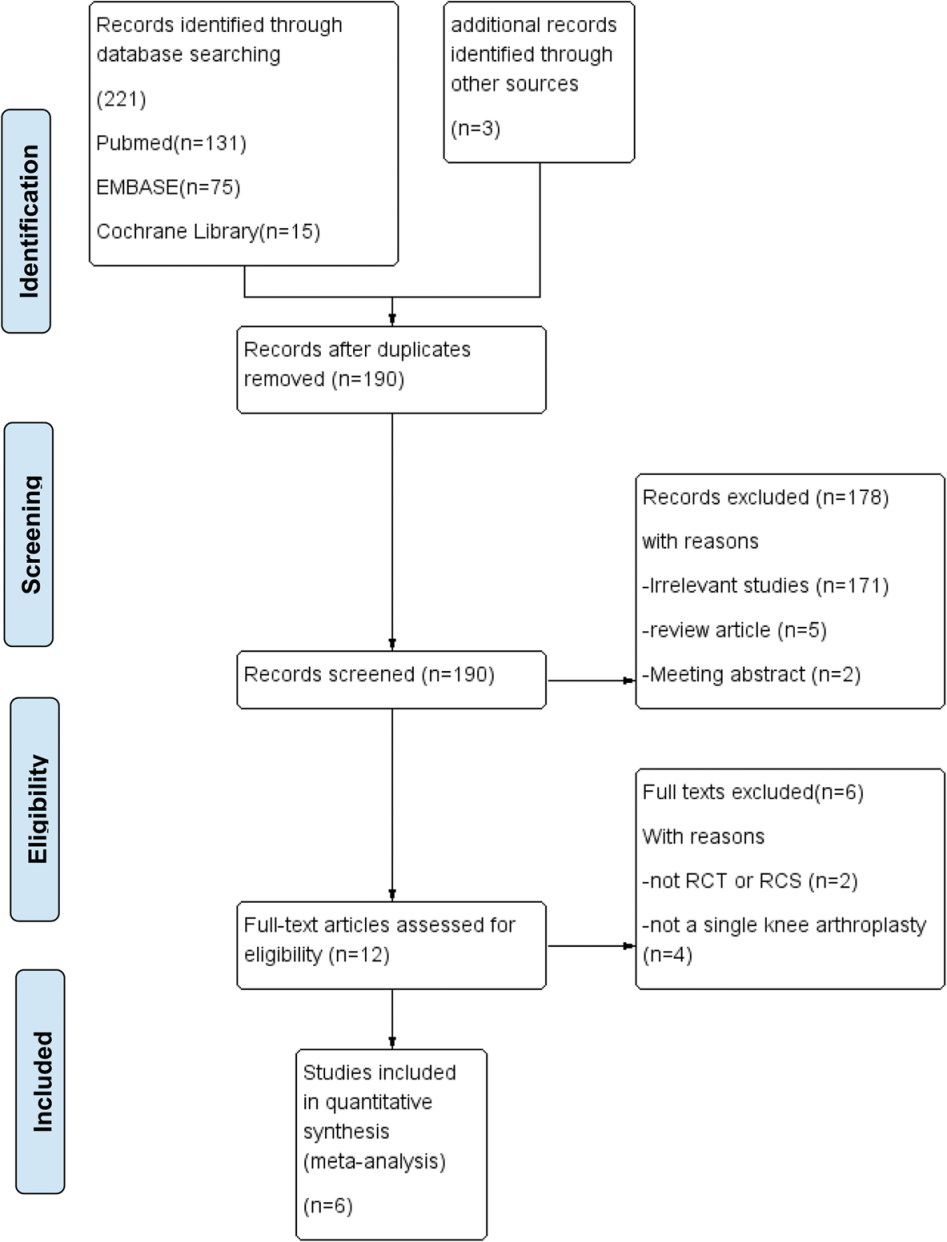
The search results and selection procedure

We systemically searched randomized controlled trials or retrospective cohort studies, including PubMed (1996–April 2017), Embase (1980–April 2017), and the Cochrane Library (CENTRAL, April 2017). Trials were also found in related references of additional studies. Only English publications were included in our meta-analysis. The search terms “total knee arthroplasty,” “total knee replacement,” “oral tranexamic acid,” and “oral TXA” were used as keywords with Boolean operators “AND” or “OR.” The search results are presented in Fig. 1.

### Inclusion criteria

Studies were eligible for the meta-analysis if they met the following PICOS (participants, intervention, comparison, outcomes, and study design) criteria: (1) participants: patients who received TKA for the first time; (2) intervention: oral administration of TXA; (3) comparison: non-TXA applied to TKA; (4) outcomes: hemoglobin drop, blood loss, transfusion rate, complications, and length of hospital stay; (5) study design: randomized controlled trials or retrospective cohort studies.

### Data extraction and quality assessment

A standard data entry form was designed for the data extraction. Two reviewers independently extracted the available study data. The extracted data included author(s), patients (*n*), publication date, age, gender, study design, body mass index, and dose of TXA. The primary outcomes were Hb drop and blood loss. The secondary outcomes consisted of the transfusion rate, length of hospital stay, and complications. We emailed the corresponding authors of studies with incomplete or graphical data. Any disagreement between two reviewers was solved by a third reviewer. The Jadad scale was used to evaluate the risk of bias for RCTs, which consisted of random sequence generation, allocation concealment, the blinding of participants and personnel, incomplete outcome data, selective reporting, and other biases [17, 18]. The study was considered high quality if the Jadad score was more than 4 points. For non-RCTs, the risk of bias was evaluated using the Newcastle-Ottawa scale [19], and a score of more than 5 points was considered high quality.

### Data synthesis

Outcomes were calculated using Windows Review Manager Software 5.3 (The Nordic Cochrane Center, The Collaboration, 2014, Copenhagen, Denmark). For continuous outcomes, we used mean difference (MD) with 95% confidence intervals (CIs) to weigh the effect interval. For noncontinuous outcomes, relative risk (RR), odds ratio (OR), or risk difference (RD) with 95% CIs were used to weigh the effect interval. The statistical heterogeneity was judged by the value of *P* and *I* ^2^ using the standard chi-squared test. *I* ^2^ > 50% and *P* < 0.1 were considered statistically significant in our results, and a random-effect model was applied for assessment; otherwise, a fixed-effect model was used for extracted data.

### Subgroup analysis

When the data had high heterogeneity, we used the random-effect model to ensure the outcome was reliable and accurate, and we performed a subgroup analysis to investigate the causes of heterogeneity. Even when the random-effect model and subgroup analysis were used to decrease the heterogeneity, there was inevitable heterogeneity in the study design and the dose of oral administration, among other factors.

## Results

### Search results

A total of 224 studies were identified through the search strategy. Thirty-four studies were excluded by Endnote software. Overall, 178 studies were excluded due to the title and abstract. According to the inclusion criteria, 6 studies were included by reading the full text: 5 were RCTs [14, 15, 20–22] and 1 was an RCS [13]. The baseline characteristics of the included studies are summarized in Tables 1 and 2.

**Table 1:** The characteristics of included studies

Oral TXA group/control group											
Studies (year)	Country	Cases	Ages (year)	Female gender (%)	Preoperative Hb (g/dL)	BMI (kg/m^2^)	Reference type	Preoperative diagnose	Preoperative thromboembolic events	Preoperative clotting disorder or anticoagulant therapy	QAS
Lee et al. (2017) [15]	China	94/95	70/68	67/69.55	13.6 ± 1.3/13.8 ± 1.2	27.7/28.4	RCT	N/A	None	None	6
Yuan et al. (2017) [14]	China	140/40	63.2/64.6	51.4/53.5	13.2 ± 0.8/13.2 ± 0.8	22.69/22.68	RCT	OA or RA	N/A	None	6
Perreault et al. (2017) [13]	USA	1049/866	66.1/66.2	62.4/58.2	14 ± 1.4/14 ± 1.4	N/A	RCS	N/A	None	N/A	6
Zohar et al. (2004) [21]	Israel	20/20	69/73	60/65	N/A	N/A	RCT	N/A	N/A	None	5
Alipour et al. (2013) [22]	Iran	27/26	68.6/63.11	59.3/56.7	N/A	28.9/29.3	RCT	OA or RA	None	None	6
Bradshaw et al. (2012) [20]	Australia	26/20	67.1/68.2	46.2/35	13.4 ± 2.5/13.8 ± 3	32.4/32.5	RCT	OA	None	None	6

*TXA* tranexamic acid, *Hb* hemoglobin, *BMI* body mass index, *QAS* quality assessment score, *RCT* randomized controlled trial, *RCS* retrospective cohort study, *N/A* not applicable, *USA* the United States of America, *OA* osteoarthritis, *RA* rheumatoid arthritis

**Table 2:** Characteristics of included studies showing general intervention information

Studies (year)	Dosage of TXA (mg)	Surgical approach	Transfusion criteria	Postoperative anticoagulation	Surgery	Anesthesia	DVT screening method	Pneumatic tourniquet
Lee et al. (2017) [15]	3000	MP	Hb < 80 g/L	N/A	Primary TKA	General anesthesia	Doppler ultrasonography	Yes
Yuan et al. (2017) [14]	N/A	MP	Hb < 80 g/L or anemia symptoms	10 mg/day rivaroxaban	Primary TKA	General anesthesia	Doppler ultrasonography and chest computed tomography	Yes
Perreault et al. (2017) [13]	3900	N/A	Hb < 80 g/L or anemia symptoms	N/A	Primary TKA	N/A	N/A	N/A
Zohar et al. (2004) [21]	3000	N/A	Hematocrit < 28%	40 mg/day enoxaparin	Primary TKA	N/A	Doppler ultrasound imaging and signs of lower limb DVT(swelling or increase in calf diameter)	Yes
Alipour et al. (2013) [22]	3000	N/A	N/A	40 mg/day enoxaparin	Primary TKA	Standard general anesthesia	N/A	Yes
Bradshaw et al. (2012) [20]	6000	MP	Hb < 70 g/L or anemia symptoms	40 mg/day enoxaparin	Primary TKA	N/A	N/A	Yes

*TXA* tranexamic acid, *N/A* not applicable, *Hb* hemoglobin, *MP* medial parapatellar approach, *DVT* deep vein thrombosis, *TKA* total knee arthroplasty

### Study characteristics

#### Risk of bias in included studies

**Fig. 2:**
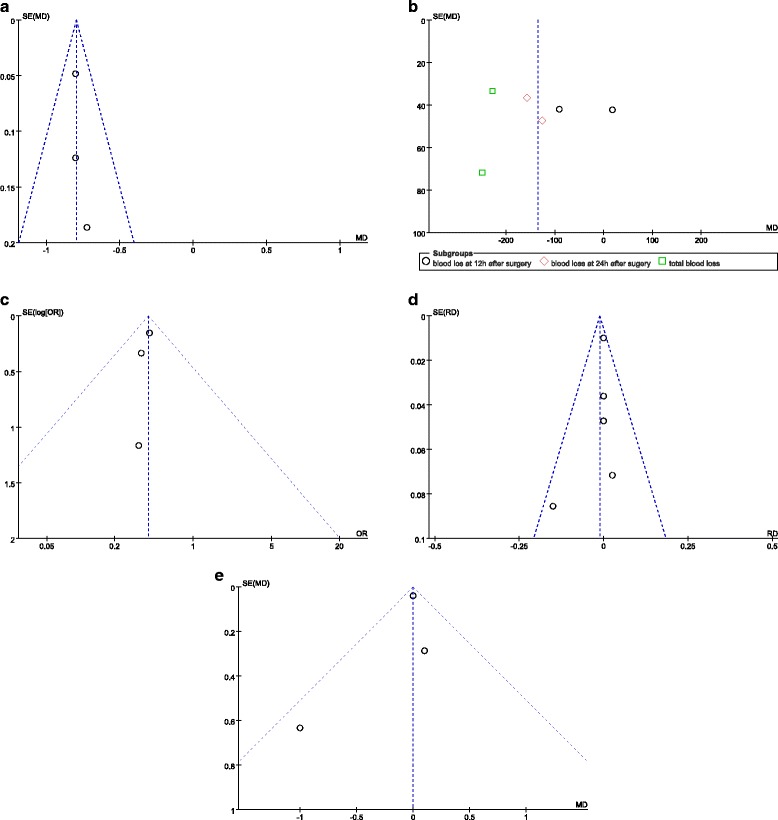
**a** A funnel plot of the Hb drop. **b** A funnel plot of the blood loss. **c** A funnel plot of the transfusion rate. **d** A funnel plot of the complications. **e** A funnel plot of the length of hospital stay

Publication bias was evaluated using a funnel plot diagram (Fig. 2). The funnel plot diagram of Hb drops, blood loss, transfusion rate, length of hospital stay, and complications was symmetrical, indicating a low risk of publication bias.

### Meta-analysis results

#### Hemoglobin drop

**Fig. 3:**
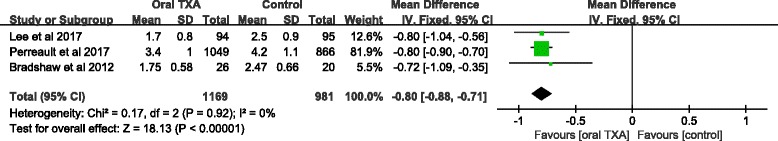
A forest plot diagram showing the Hb drop (g/dL)

Data from three studies including 2150 patients reported the Hb drops at the lowest postoperative Hb level during hospital stays. Compared to the control group, oral TXA significantly prevented Hb drop (MD = − 0.80; 95% CI, − 0.88, − 0.71; *P* < 0.05; Fig. 3). The fixed-effect model was used to find the statistical heterogeneity between the two groups (*x* ^2^ = 0.17; df = 2; *P* = 0.92; *I* ^2^ = 0%; Fig. 3).

#### Blood loss

**Fig. 4:**
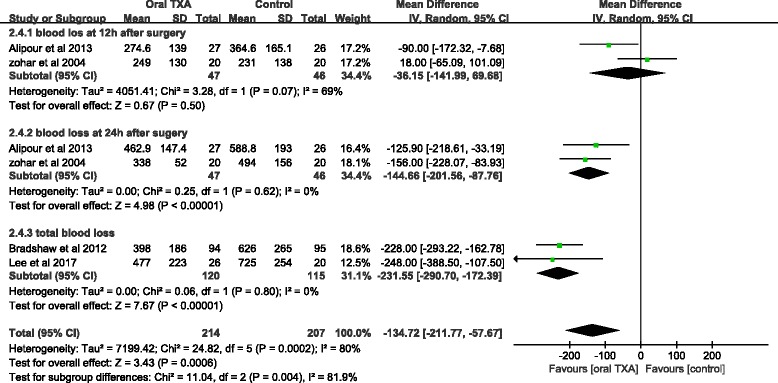
A forest plot diagram showing the blood loss (mL)

The blood loss 12 h after surgery was reported in two studies totaling 93 patients. No significant difference was found between the oral TXA and the control groups (MD = − 36.15; 95% CI, − 141.99, 69.68; *P* > 0.05; Fig. 4). The blood loss 24 h after surgery was reported in two studies of 93 patients. Compared to the control groups, oral TXA significantly prevented blood loss 24 h after surgery (MD = − 144.66; 95% CI, − 201.56, − 87.76; *P* < 0.05; Fig. 4). Data from two studies including 235 patients recorded the total blood loss. The oral TXA groups had significantly less blood loss compared to the control groups (MD = − 231.55; 95% CI, − 290.70, − 172.39; *P* < 0.05; Fig. 4). Statistical heterogeneity was not found in blood loss 24 h after surgery (*x* ^2^ = 0.25; df = 1; *P* = 0.62; *I* ^2^ = 0%) or total blood loss (*x* ^2^ = 0.06; df = 1; *P* = 0.80; *I* ^2^ = 0%; Fig. 4). A random-effects model was applied in the study due to the statistical heterogeneity found in blood loss 12 h after surgery (*x* ^2^ = 3.28; df = 1; *P* = 0.07; *I* ^2^ = 69%; Fig. 4).

#### Transfusion rate

**Fig. 5:**
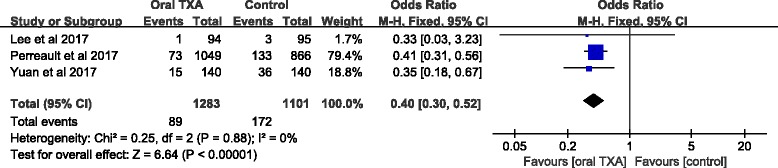
A forest plot diagram showing the transfusion rate (%)

The transfusion rate was reported in three studies including 2384 patients. Compared to the control groups, the oral TXA groups had significantly reduced transfusion rates (OR = 0.40; 95% CI, 0.30, 0.52; *P* < 0.05; Fig. 5). A fixed-effect model was applied because no statistical heterogeneity was found in this meta-analysis (*x* ^2^ = 0.25; df = 2; *P* = 0.88; *I* ^2^ = 0%; Fig. 5).

#### Complications

**Fig. 6:**
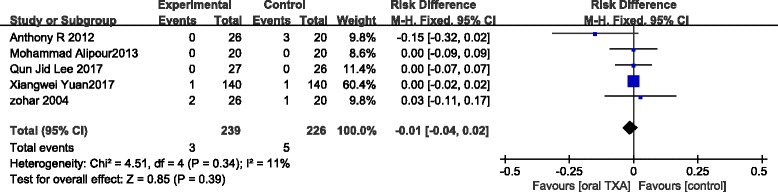
A forest plot diagram showing the complications (%)

We extracted data on complications including deep vein thrombosis (DVT) and pulmonary embolism (PE) from five studies including 465 patients. There was no significant difference between the oral TXA and the control groups (RD = − 0.01; 95% CI, − 0.04, 0.02; *P* > 0.05; Fig. 6). Because of the low statistical heterogeneity, we used a fixed-effect model (*x* ^2^ = 4.51; df =4; *P* = 0.34; *I* ^2^ = 11%; Fig. 6).

#### Length of hospital stay

**Fig. 7:**
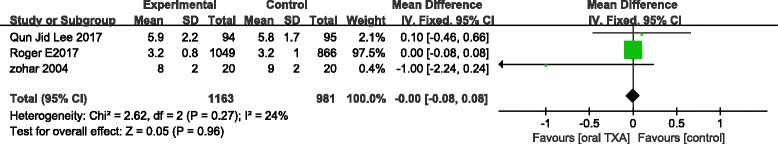
A forest plot diagram showing the length of hospital stay (days)

Length of hospital stay was reported in three studies involving 2144 patients. No significant difference was found between the two groups (MD = − 0.00; 95% CI, − 0.08, 0.08; *P* > 0.05; Fig. 7). Because of the low statistical heterogeneity, we used a fixed-effect model (*x* ^2^ = 2.62; df = 2; *P* = 0.27; *I* ^2^ = 24%; Fig. 7).

## Discussion

Recently, attention has been paid to oral TXA due to its rapid absorption, operability, and less cost [23, 24]. Applicable management during the perioperative period reduces both the recovery time and the transfusion rate. TXA as one of the most important antifibrinolytics has shown strong effects on reducing blood loss by blocking the lysine-binding sites of plasminogen. The two methods of topical application and intravenous injection administration prevented significant blood loss [25–27]. Anaphylactic shock was reported in patients using intravenous injection, and there was also a potential risk of periprosthetic infection once the needle was contaminated [11, 12]. It has recently been reported that oral TXA has the same effects in preventing blood loss, Hb drop, and the transfusion rate compared to intravenous or topical administration [28].

In our meta-analysis, all patients in the included studies had been diagnosed without preoperative coagulation disorders. Alipour et al. [22] excluded from the study patients with thromboembolic events and those who had recently taken anticoagulants or non-steroidal anti-inflammatory drugs. Bradshaw et al. [20] had exclusion criteria that included a history of thromboembolic events and/or anticoagulation that could not be ceased within the recommended time before surgery. Patients with thromboembolic diseases, anticoagulation, or DVT prophylaxis were excluded by Lee et al [15]. Yuan et al. [14] excluded patients with coagulopathy or bleeding disorders. Zohar et al. [21] excluded patients with bleeding disorders or current anticoagulant therapy. Perreault et al. [13] excluded patients with a history of pulmonary embolus or DVT. Three of the included studies recorded preoperative hemoglobin levels. Lee et al. [15] reported preoperative Hb levels in the oral and control groups of 13.3 ± 1.3 g/dL and 13.8 ± 1.2 g/dL, respectively. Yuan et al. [14] reported preoperative Hb levels of 13.2 ± 0.8 g/dL and 13.2 ± 0.8 g/dL, respectively, in the oral and control groups. Bradshaw et al. [20] reported preoperative Hb levels of 13.4 ± 2.5 g/dL and 13.8 ± 3 g/dL in the oral and control groups, respectively. Perreault et al. [13] reported preoperative Hb levels of 14.1 ± 1.4 g/dL in the oral group and 14 ± 1.4 g/dL in the control group.

Concerning the surgical approach, the medial parapatellar method was performed by Lee et al. [15], Yuan et al. [14], and Bradshaw et al. [20]. Three of the included studies did not record the surgical methods [13, 21, 22]. Bradshaw et al. [20] reported that the indications for blood transfusion were hemoglobin levels of less than 7 g/dL or clinical symptoms of anemia. Lee et al. [15] recorded that the serum Hb level for transfusion trigger was 8 g/dL. Yuan et al. [14] showed that blood transfusions were performed following a restrictive strategy (Hb < 8 g/dL) or symptoms of anemia. Zohar et al. [21] reported that hematocrit < 28% constituted the postoperative transfusion trigger. Perreault et al. [13] settled on a predefined protocol, with transfusion of Hb < 8 g/dL or Hb < 10 g/dL in patients with significant symptoms of anemia.

Concerning the anticoagulation method, rivaroxaban (10 mg/day) was taken orally until 21 days after hospital discharge in Yuan et al.’s research [14]. In Zohar et al.’s [21] study, subcutaneous enoxaparin (40 mg/day) was administered for DVT prophylaxis. Alipour et al. [22] reported that enoxaparin with a dose of 40 mg/day was prescribed from 1 day before surgery and maintained for 2 weeks. Bradshaw et al. [20] reported that anticoagulation consisted of 40 mg/day of enoxaparin was administered for 2 weeks starting at 12 h postoperatively. Lee et al. [15]reported all patients had Doppler ultrasonography on postoperative day 7 to detect any proximal DVT. In Zohar et al.’s [21] trial, all patients were examined daily for signs of lower limb DVT (swelling or an increase in the calf diameter) and underwent lower limb Doppler ultrasound imaging on the fifth postoperative day.

Hb drop was the primary outcome in our meta-analysis. Several high-quality RCTs and RCSs showed that oral TXA can prevent Hb drop postoperatively [13, 15]. Perreault et al. [13] found that there was less Hb drop in the oral TXA group compared to the untreated group. These results were consistent with our outcomes. Our pooled data demonstrated that oral TXA can prevent Hb drop postoperatively. Irwin et al. [29] demonstrated that oral TXA provided similar effects on lower Hb drop compared to intravenous TXA. In conclusion, oral TXA can effectively inhibit Hb drop.

Blood loss was also an important indicator for evaluating blood sparing. An RCT reported by Bradshaw et al. [20] showed that oral TXA prevented total blood loss significantly compared to the untreated group, and the same outcome was reported by Lee et al. [15]. Our meta-analysis was consistent with these results. In our meta-analysis, oral TXA significantly reduced total blood loss compared to the control group. Two high-quality RCTs showed that oral TXA could limit blood loss 24 h after surgery [21, 22]. This is consistent with our findings. As to blood loss at 12 h after surgery, Zohar et al. [21] reported that the amount of blood loss in the surgical drain was similar in the oral TXA group and the control group. This is also consistent with our findings. Our meta-analysis concluded that no significant differences were found between the oral TXA group and the control group. Meanwhile, risk of bias and high heterogeneity should be considered when interpreting the findings. We considered that the imbalance between fibrinolysis and TXA-induced antifibrinolytics is due to the release of tourniquet. Tourniquet release can accelerate fibrinolysis, while the optimal effects of oral TXA appeared 2 h after ingestion [16, 30]. As a consequence, the oral TXA group did not have inhibited fibrinolysis caused by tourniquet release. Therefore, no significant differences in blood loss were found in the oral and control TXA groups 12 h after surgery. However, because of the small sample size (under 100 patients), the conclusion with respect to blood loss was limited compared to the findings on Hb drop and transfusion rate.

The blood-sparing efficacy was also reflected in the transfusion rate. Perreault et al. [13] used a large hospital database to assess the rate of transfusion among the oral TXA and control groups. The outcomes of this RCT demonstrated that the oral TXA group had a lower rate of transfusion compared to the control group. This is consistent with our findings. Lee et al. [15] reported that there were no significant differences in transfusion rates between the two groups. The possible reason for low transfusion rate in both groups was the high postoperative Hb level compared to the transfusion trigger [31]. Thus, we conclude that the oral TXA group has a lower transfusion rate compared to the control group. Postoperative DVT and PE were common complications in TKA patients who took TXA. Our meta-analysis failed to find any significant differences between the two groups. Similar findings were reported by Lee et al. [15]. The results of our pooled data indicated that there were no differences in length of hospital stay because of the low incidence in both groups. Taking these findings together, we considered that there were no obvious differences in complications and length of hospital stay in the oral TXA group and the control group. Moreover, it has been reported that the cost of oral TXA administration was much lower compared to intravenous or topical forms [14, 32, 33]. However, all patients of our included studies have been diagnosed without history of thromboembolic events or a higher thromboembolic risk, and there was less or even no data available dealing with the use of TXA with a higher thromboembolic risk. Taking these into consideration, it may potentially limit the general use of TXA in elective surgeries.

Several reports have demonstrated that oral TXA helps reduce hematocrit drop [20, 22]. Our meta-analysis did not analyze hematocrit drop for two reasons: because of the small sample of included studies, and the ratio between Hb drop and hematocrit drop is always supposed to be 1/3. Patients with potential malfunctioning coagulation systems such as hemophilia, unknown bleeding disorders, active liver disease, and so on usually cannot be detected without preoperative diagnostics during preoperative blood management, while malfunctioning coagulation system can potentially cause postoperative DVT [34]. In our meta-analysis, Lee et al. [15] excluded 46 patients who had potentially malfunctioning coagulation systems and a history of thromboembolic disease and other disease. Yuan et al. [14] excluded three patients with clotting mechanism disorders throughout preoperative diagnostics. Remaining studies failed to carry out preoperative diagnostics on patients during preoperative blood management, which may increase the risk of thrombus.

Our meta-analysis and systematic review have several limitations: (1) Only five studies were included in our meta-analysis. The statistical results would be better if more RCTs had been included. (2) Outcomes such as hematocrit and cost were not analyzed due to the small sample size and/or insufficient data. (3) Only English publications were included in our meta-analysis, so there is publication bias. (4) Because of the significant heterogeneity of blood loss at 12 h (*I* ^2^ = 69%), we attempted to investigate the source of heterogeneity. Throughout the full articles, the administrations time of TXA before surgery were different (1 and 2 h before surgery, respectively) and the length of enoxaparin use showed large differences (1 and 14 days, respectively) in two studies. Considering the short half-life of oral TXA and the anticoagulant effect of enoxaparin, we believe these differences were the major sources of the heterogeneity. Meta-analyses (PRISMA) guidelines and the Cochrane Handbook were used to assess the quality of the data published in the included studies to ensure that the results of our meta-analysis were reliable and receivable.

## Conclusions

Compared to the control group, Hb drop, blood loss, and transfusion rates can be significantly reduced using oral TXA without increasing the risk of complications. Moreover, oral administration is cheaper and easier to use compared to IV and topical applications. Nevertheless, oral TXA should be restricted to selected patients after adequate preoperative blood management and surgeries with extensive blood loss, as there is no current clear recommendation for the general use of TXA in elective orthopedic surgeries such as TKA.

## Abbreviations

CIs: Confidence intervals
DVT: Deep vein thrombosis
Ha: Hematocrit
Hb: Hemoglobin
LOS: Length of stay
MD: Mean difference
MP: Medial parapatellar approach
OA: Osteoarthritis
OR: Odds ratio
PE: Pulmonary embolism
PRISMA: Preferred Reporting Items for Systematic Review and Meta-Analyses
QAS: Quality assessment score
RA: Rheumatoid arthritis
RCSs: Retrospective cohort studies
RCTs: Randomized controlled trials
RD: Risk difference
RR: Relative risk
TKA: Total knee arthroplasty
TXA: Tranexamic acid

## References

[1] Gidwani S, Fairbank A (2004). The orthopaedic approach to managing osteoarthritis of the knee. BMJ.

[2] Yang ZG, Chen WP, Wu LD (2012). Effectiveness and safety of tranexamic acid in reducing blood loss in total knee arthroplasty: a meta-analysis. J Bone Joint Surg Am.

[3] Alshryda S (2014). A systematic review and meta-analysis of the topical administration of tranexamic acid in total hip and knee replacement. Bone Joint J.

[4] Poeran J (2014). Tranexamic acid use and postoperative outcomes in patients undergoing total hip or knee arthroplasty in the United States: retrospective analysis of effectiveness and safety. BMJ.

[5] Chen TP (2017). Comparison of the effectiveness and safety of topical versus intravenous tranexamic acid in primary total knee arthroplasty: a meta-analysis of randomized controlled trials. J Orthop Surg Res.

[6] Li JF (2017). Combined use of intravenous and topical versus intravenous tranexamic acid in primary total knee and hip arthroplasty: a meta-analysis of randomised controlled trials. J Orthop Surg Res.

[7] Mi B (2017). Is combined use of intravenous and intraarticular tranexamic acid superior to intravenous or intraarticular tranexamic acid alone in total knee arthroplasty? A meta-analysis of randomized controlled trials. J Orthop Surg Res.

[8] Alshryda S (2011). Tranexamic acid in total knee replacement: a systematic review and meta-analysis. J Bone Joint Surg Br.

[9] Panteli M (2013). Topical tranexamic acid in total knee replacement: a systematic review and meta-analysis. Knee.

[10] Lucaspolomeni MM (2004). A case of anaphylactic shock with tranexamique acid (Exacyl). Ann Fr Anesth Reanim.

[11] Klak M (2010). Tranexamic acid, an inhibitor of plasminogen activation, aggravates staphylococcal septic arthritis and sepsis. Scand J Infect Dis.

[12] Zhang LK, et al. Comparison of oral versus intravenous application of tranexamic acid in total knee and hip arthroplasty: A systematic review and meta-analysis. Int J Surg. 2017;45:77–84. 10.1016/j.ijsu.2017.07.097 28755884

[13] Perreault RE, et al. Oral Tranexamic Acid Reduces Transfusions in Total Knee Arthroplasty. J Arthroplasty. 2017;32(10):2990–4. 10.1016/j.arth.2017.03.063 28757131

[14] Yuan X, et al. Comparison of 3 Routes of Administration of Tranexamic Acid on Primary Unilateral Total Knee Arthroplasty: A Prospective, Randomized, Controlled Study. J Arthroplasty. 2017;32(9):2738–43. 10.1016/j.arth.2017.03.059 28455182

[15] Lee QJ, Ching WY, Wong YC (2017). Blood sparing efficacy of oral tranexamic acid in primary total knee arthroplasty: a randomized controlled trial. Knee Surg Relat Res.

[16] Beckett AH, Taylor JF, Kourounakis P (1970). The absorption, distribution and excretion of pentazocine in man after oral and intravenous administration. J Pharm Pharmacol.

[17] Hartling L (2009). Risk of bias versus quality assessment of randomised controlled trials: cross sectional study. BMJ.

[18] Jadad AR (1996). Assessing the quality of reports of randomized clinical trials: is blinding necessary?. Control Clin Trials.

[19] Wells GA (2000). The Newcastle–Ottawa scale (NOS) for assessing the quality of non-randomized studies in meta-analysis. Appl Eng Agric.

[20] Bradshaw AR (2012). Oral tranexamic acid reduces blood loss in total knee replacement arthroplasty. Current Orthopaedic Practice.

[21] Zohar E (2004). The postoperative blood-sparing efficacy of oral versus intravenous tranexamic acid after total knee replacement. Anesth Analg.

[22] Alipour M (2013). Effectiveness of oral Tranexamic acid administration on blood loss after knee artroplasty: a randomized clinical trial. Transfus Apher Sci.

[23] Fillingham YA (2016). The James A. Rand Young Investigator's Award: a randomized controlled trial of oral and intravenous Tranexamic acid in total knee arthroplasty: the same efficacy at lower cost?. J Arthroplast.

[24] Lee QJ, Chang WY, Wong YC (2017). Blood-sparing efficacy of oral tranexamic acid in primary total hip arthroplasty. J Arthroplast.

[25] Wang C (2015). Topical application of tranexamic acid in primary total hip arthroplasty: a systemic review and meta-analysis. Int J Surg.

[26] Roy SP (2012). Efficacy of intra-articular tranexamic acid in blood loss reduction following primary unilateral total knee arthroplasty. Knee Surg Sports Traumatol Arthrosc.

[27] Konig G, Hamlin BR, Waters JH (2013). Topical tranexamic acid reduces blood loss and transfusion rates in total hip and total knee arthroplasty. J Arthroplast.

[28] Mcgrath S, Yates P, Prosser G (2014). Oral tranexamic acid in hip and knee arthroplasty: a prospective cohort study. Open J Orthopedics.

[29] Irwin A (2013). Oral versus intravenous tranexamic acid in enhanced-recovery primary total hip and knee replacement: results of 3000 procedures. Bone Joint J.

[30] Tanaka N (2001). Timing of the administration of tranexamic acid for maximum reduction in blood loss in arthroplasty of the knee. J Bone Joint Surg Br.

[31] Engel JM (2001). Regional hemostatic status and blood requirements after total knee arthroplasty with and without tranexamic acid or aprotinin. Anesth Analg.

[32] Moskal JT, Harris RN, Capps SG (2015). Transfusion cost savings with tranexamic acid in primary total knee arthroplasty from 2009 to 2012. J Arthroplast.

[33] Gillette BP (2013). Economic impact of tranexamic acid in healthy patients undergoing primary total hip and knee arthroplasty. J Arthroplast.

[34] Mont MA (2011). Preventing venous thromboembolic disease in patients undergoing elective hip and knee arthroplasty. J Am Acad Orthop Surg.

